# Gating Movement of Acetylcholine Receptor Caught by Plunge-Freezing

**DOI:** 10.1016/j.jmb.2012.07.010

**Published:** 2012-10-05

**Authors:** Nigel Unwin, Yoshinori Fujiyoshi

**Affiliations:** 1 MRC Laboratory of Molecular Biology, Hills Road, Cambridge CB2 0QH, UK; 2 Department of Biophysics, Faculty of Science, Kyoto University, Oiwake, Kitashirakawa, Sakyo-ku, Kyoto 606‐8502, Japan

**Keywords:** ACh, acetylcholine, AChBP, acetylcholine-binding protein, FSC, Fourier shell correlation, CMS, congenital myasthenic syndrome, PDB, Protein Data Bank, acetylcholine receptor, freeze-trapping, asymmetric gating, allosteric mechanism, electron microscopy

## Abstract

The nicotinic acetylcholine (ACh) receptor converts transiently to an open-channel form when activated by ACh released into the synaptic cleft. We describe here the conformational change underlying this event, determined by electron microscopy of ACh-sprayed and freeze-trapped postsynaptic membranes. ACh binding to the α subunits triggers a concerted rearrangement in the ligand-binding domain, involving an ~ 1‐Å outward displacement of the extracellular portion of the β subunit where it interacts with the juxtaposed ends of α-helices shaping the narrow membrane-spanning pore. The β-subunit helices tilt outward to accommodate this displacement, destabilising the arrangement of pore-lining helices, which in the closed channel bend inward symmetrically to form a central hydrophobic gate. Straightening and tangential motion of the pore-lining helices effect channel opening by widening the pore asymmetrically and increasing its polarity in the region of the gate. The pore-lining helices of the α_γ_ and δ subunits, by flexing between alternative bent and straight conformations, undergo the greatest movements. This coupled allosteric transition shifts the structure from a tense (closed) state toward a more relaxed (open) state.

## Graphical Abstract

**Figure:**
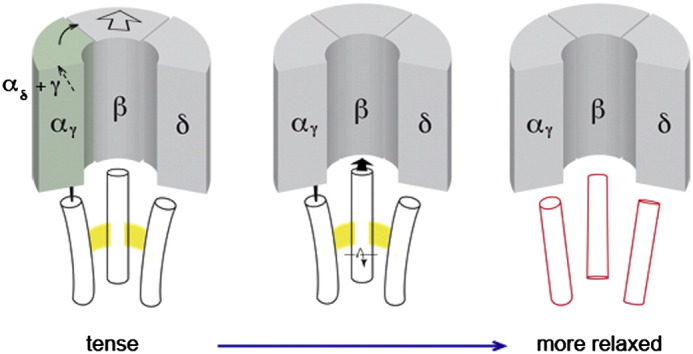

**Research Highlights:**

► Effect of acetylcholine at binding site is communicated to the gate ~50Å away. ► The structural change has been analysed by spray-freeze-trapping electron microscopy. ► A small rearrangement in the extracellular domain pushes the β subunit outward. ► This destabilises the closed-channel configuration of membrane helices. ► Channel opens mainly by flexure of pore-lining helices of the αγ and δ subunits.

## Introduction

The nicotinic acetylcholine (ACh) receptor is the ligand-gated ion channel mediating fast chemical transmission of electrical signals between nerve cells and muscle cells. It is the best characterised member of a family of pentameric ion channels (the Cys-loop family^1–3^), including neuronal ACh receptors, sharing rapid gating properties and a conserved molecular design. Like most other family members, the muscle-type ACh receptor is a heteropentamer composed of subunits having similar or identical amino acid sequences arranged in a ring around a narrow membrane pore. ACh binds to this assembly at sites projecting far into the synaptic cleft, whereas the gate is located in the membrane more than 50 Å away.

A fundamental question concerning the function of the ACh receptor is how is the effect of ligand binding communicated almost instantly over such a large distance to open the gate and create an ion-conducting pore? The subunits (α_γ_, β, δ, α_δ_ and γ) are long rods divided into distinct parts (an N-terminal extracellular portion organised around a β-sandwich core, a membrane-spanning portion composed of four α-helices and an intracellular portion containing one α-helix) to enable this event.^4,5^ ACh binds to α_γ_ and α_δ_ at the interface with neighbouring γ and δ subunits,^6^ and the pore-lining helices of the closed channel make a hydrophobic constriction in the membrane, which functions as the gate.^3,4,7^ Hence, the conformational change to open the channel is initiated by ACh binding to the β-sandwich portion and must be transmitted through connecting loops to the membrane helices, switching them to an alternative configuration that is permeable to ions.

A number of approaches have yielded insight into the nature of this conformational change.^7–12^ X-ray structures of related homopentameric ion channels, solubilised in detergent, have illuminated differences between possible functional states.^13–16^ However, there are as yet no precise structural data giving details of the physiological transition in the receptor itself. A previous electron crystallographic study of the receptor in the closed-channel form,^5^ and comparison with a related soluble pentameric ACh-binding protein (AChBP),^17–19^ had hinted how relative movements of the inner and outer β-sheets of the α subunits might initiate channel opening by coupling to the ends of α-helices lining the membrane-spanning pore. Nevertheless, these movements have not been observed directly; neither is it known how in response to such movements the individual helices change their orientation and shape.

Here, we investigate the structural mechanism of ACh-receptor gating by propelling ACh-containing spray droplets onto postsynaptic membrane vesicles lying within a thin aqueous film and then trapping the reaction by rapid freezing.^8,20^ The vesicles used, from the (muscle-derived) electric organ of the *Torpedo* ray, are tubes having receptors on a helical surface lattice,^21,22^ where the membrane curvature and molecular packing are like that at the crests of the junctional folds.^23^ The tubes are imaged at 4K^24^ in the frozen film and analysed at ~ 6 Å resolution by electron crystallography. By such means, we recapitulate the brief reaction of neurotransmitter with receptors at the synapse and determine the structure of the opened channels in a physiological ionic environment and native membrane setting.

Direct imaging of the receptor in the intact membrane, following synaptic-like activation, enables its gating movement to be evaluated with precision and unambiguously compared with other structural approaches that require perturbing influences such as the presence of detergent or recombinant modification of the polypeptide chain. We show that the subunit harbouring the low-affinity ACh binding site, α_γ_, plays the primary role in driving the extended conformational change, whereas the β subunit plays the primary role in communicating this change to the membrane. The channel opens by asymmetric motions of the pore-lining helices.

## Results

### Freeze-trapping

**Fig. 1:**
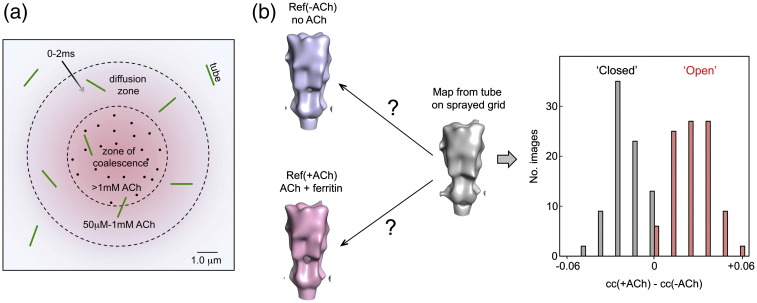
ACh-spray activation and identification of tubes containing open channels. (a) Mixing of a 1‐μm‐diameter spray droplet, containing ACh and ferritin, with the grid-supported aqueous film 10 ms after impact: ACh (reddish colour) spreads beyond the ferritin (dots) within the zone of coalescence (inner dashed circle), reaching some tubes by diffusion; based on Ref. 20. (b) Sorting of ‘unlabelled’ tubes into closed and open classes by pairwise comparison of single-image density maps with reference maps, Ref(+ ACh) and Ref(− ACh), obtained from ACh-exposed ferritin-labelled tubes and from untreated tubes, respectively. The histogram plots the number of images against the difference between the two correlation coefficients (see Methods).

Synaptic activation of muscle-type receptors by ACh is highly efficacious, with at least 90% of the channels being open initially when exposed to saturating amounts (greater than ~ 50 μM; Refs. 25–27). To achieve similar efficacy *in vitro*, with minimal desensitisation,^27^ we used an atomiser spray coupled to a plunge-freezing device.^20^ Solutions containing the tubes were applied to electron microscope grids, which were blotted to produce thin aqueous films and plunged by free fall into liquid-nitrogen‐cooled ethane. Spray droplets, containing 100 mM ACh and ferritin marker particles, were made to impinge on the grids 10 ms before they hit the ethane surface, thus allowing brief mixing with the film contents before freezing (Fig. 1a). Tubes within the frozen films were photographed in areas inside the coalescing droplets (identified by the presence of ferritin) and also beyond their ferritin-delineated edges, where the ACh concentration may have reached saturating amounts by diffusion.^20^ In the images, therefore, ferritin-marked tubes should contain mainly open channels, whereas the others should contain mainly open or mainly closed channels—depending on whether the ACh concentration had reached saturating amounts.

### ACh-induced structural change

To evaluate the structural change induced by ACh, we reconstructed from each image a low‐resolution three-dimensional density map (Methods; see also Table 1). Initially, two reference maps were built by averaging these single-image maps: one [Ref(+ ACh)] from tubes within the zone of coalescence (Fig. 1a), where the local ACh concentration was 1–5 mM (estimated from the number of nearby ferritin particles), while the other [Ref(− ACh)] from tubes on unsprayed grids. When the densities composing Ref(− ACh) were subtracted from Ref(+ ACh), differences appeared in the vicinity of the ACh binding region of the α_γ_ subunit and at the outer surface of β next to the δ subunit. A statistical ‘*t*-map’^28^ indicating the significance of these differences (Fig. S1) confirmed that they were reproducible and, hence, that an ACh-induced structural change could be detected reliably in single tubes at low resolution.

**Fig. 2:**
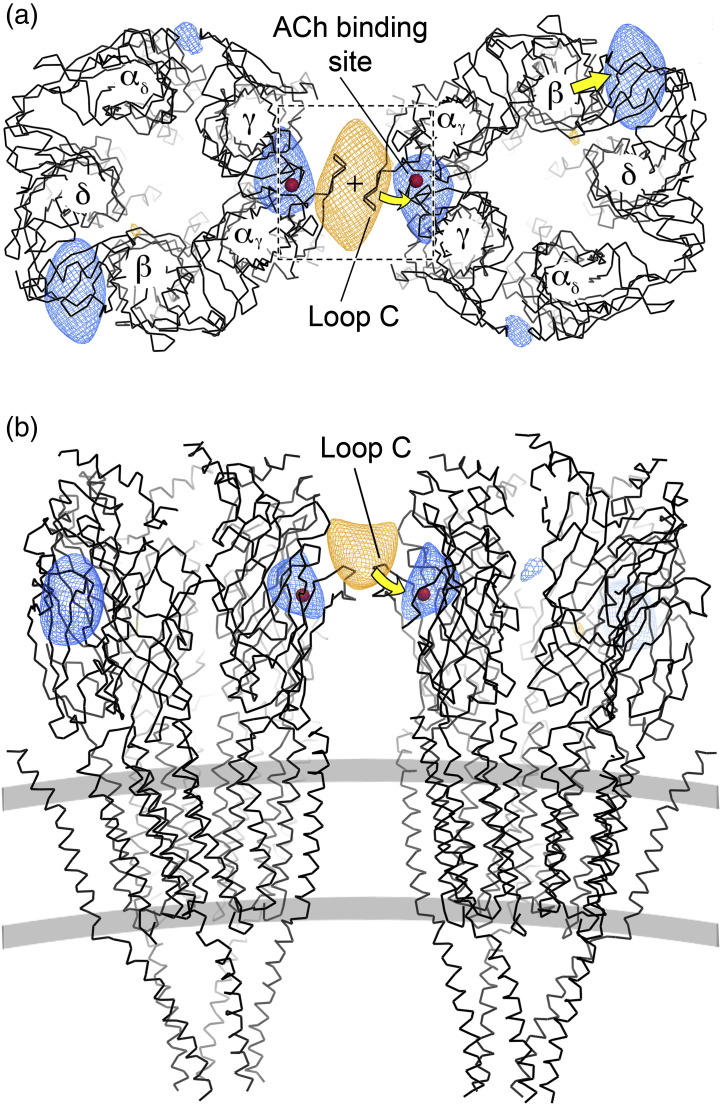
Reproducible structural changes induced by ACh activation. Regions of most significant difference between the closed- and open-class density maps are superimposed on a pair of neighbouring receptors^5^ (PDB ID: 2BG9) docked into the tube surface lattice. Views are (a) from the synaptic cleft and (b) parallel to the membrane (grey bars). The locations of individual polypeptide chains within each receptor^5,19^ are indicated. Dashed box in (a), area shown in Fig. 3a; central cross, radial 2-fold axis; red spheres, ACh binding site in α_γ_. Contours at *P* = 0.001: orange indicates decrease and blue indicates increase in density on exposure to ACh; yellow arrows indicate the nature of the structural displacements giving rise to the probability peaks.

Next, we evaluated images from the sprayed grids where ferritin was absent, but where a fraction of the tubes may have received saturating concentrations of ACh because of their proximity to a coalescing droplet. Most such images yielded density maps correlating well with one but not the other reference map (typically ~ 3% difference), while the remaining ~ 10% gave weak or no correlation preference, as if they contained similar numbers of closed and open channels. Figure 1b shows the bimodal histogram arising from the pairwise comparisons, when plotted in terms of differences in correlation coefficient [cc(+ ACh) − cc(− ACh)]. Division of these images into ‘closed’ and ‘open’ classes according to correlation preference, and averaging of the sorted maps to represent each class, led to an equivalent ‘*t*-map’ (Fig. 2) as was achieved previously from a smaller number of images with ACh either absent or present in (roughly) known amounts. Hence, that original structural change occurred also in the open-class tubes, where the maximum ACh concentration was only ~ 1 mM and the reaction time extremely brief (0–2 ms; Fig. S2). Such an ‘all-or-none’ response, over the wide ACh concentration range and timescale used, is consistent with the channel-opening properties indicated by electrophysiological measurement.^25–27^

### Closed- and open-channel structures

Only the most substantial ACh-induced changes are evident at low resolution (Fig. 2). They involve a movement inward of a loop (loop C) at the ACh binding site of α_γ_ (curved yellow arrows) and an outward displacement of the extracellular portion of the β subunit (straight yellow arrow). To obtain a more detailed description of this conformational change, we determined structures from the two classes to 6.2 Å resolution [Fourier shell correlation (FSC)_0.5_ criterion, Fig. S3], by averaging the complete data from all images in each class. As Table 1 shows, just over 100 images were used for both structures, and 77% of the open-class images were from tubes lying beyond the zone of coalescence, where the ACh exposure would have reproduced near-perfectly conditions at a nerve–muscle synapse.^31^

**Fig. 3:**
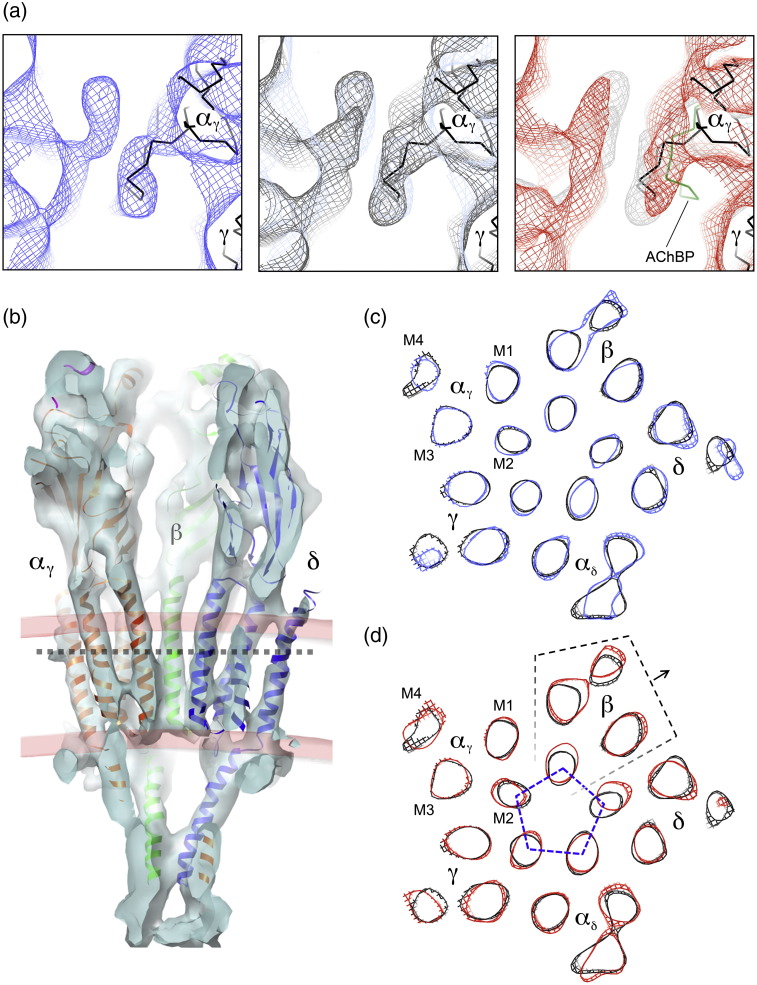
Loop-C conformation and pore sizes of closed- and open-class structures. (a) Boxed area in Fig. 2 showing densities from the three ~ 6‐Å maps (blue, untreated tubes; grey, closed class; red, open class), and superimposed C^α^ backbones from the atomic model^5^ (black, PDB ID: 2BG9) and (right) loop C from aligned AChBP^18^ (green; PDB ID: 1I9B). (b) Central slab through ACh receptor, showing densities from the closed-class structure, and superimposed C^α^ backbone from the atomic model (ribbon representation; α, β, γ and δ in red, green, magenta and blue, respectively). The phospholipid headgroup regions (pink contours) are identified by two prominent bands of density running concentrically about the axis of the tube;^22,29^ their locations coincide with rings of negatively charged amino acids that affect conductance through the open pore.^30^ (c and d) Comparison of closed- and open-channel densities from the three ~ 6‐Å maps in a cross section through the extracellular leaflet of the lipid bilayer [level of dashed bar in (b)]; colours as in (a). The broken lines in (d) highlight the pentagonally symmetric arrangement of M2 helices around the pore of the closed channel and the movement outwards of all four helices of β when the channel opens. The mesh interval corresponds to 1 Å; all contours at 1 σ.

For comparison, we also obtained a structure to a similar resolution (FSC_0.5_: 6.5 Å; Fig. S3) from tubes used earlier to derive an atomic model of the ACh receptor.^5^ These tubes were not exposed to ACh, and have different helical symmetry from the present ones (Table 1), and so give an independent representation of the receptor in the closed-channel form. We find that the structure from the closed-class tubes has a similar loop-C conformation (Fig. 3a, left and middle), the same pore profile (Fig. 3b) and the same pore cross section (Fig. 3c) as the structure from the unexposed tubes. In terms of these defining criteria, there is no distinction, confirming that the sorting procedure was effective in selecting tubes where most channels are closed. In contrast, the open-class structure shows loop C to be bent further into the binding pocket (red compared with grey contours in Fig. 3a, right) and the pore to be wider (red contours in Fig. 3d), as required for the open-channel form of the receptor. It will be described later how the pore widens, especially in the extracellular leaflet of the bilayer, through asymmetric motions of the pore-lining M2 helices and movement of all four helices composing the β subunit.

Loop C is better resolved in α_γ_ than in α_δ_ in the closed-channel structure,^5^ presumably because its flexibility is restricted by interactions with the equivalent loop in a neighbouring receptor. While these interactions are not identical in the two closed-channel maps (due to differences in lattice curvature), loop C in both examples (Fig. 3a, left and middle) extends tangentially from the body of α_γ_, creating an entry path for ACh at the α_γ_–γ interface. The new orientation adopted by loop C in the open-class structure (Fig. 3a, right) is intermediate between that of the closed channel and that of loop C in ligand-bound AChBP,^17,18^ which may reflect a fully coordinated desensitised state.^32^

In addition to the local disturbance around loop C, we find specific differences between the closed- and open-class maps, indicating that ACh drives a more extended change in the extracellular part of the α subunits. This in turn affects other subunits in the ligand-binding domain, bringing changes to the membrane domain and, consequently, to the gate. While it is possible to view the observed changes in terms of a ‘conformational wave’,^10^ or in terms of an assembly that simply converts from one preexisting conformation to another,^33^ we prefer to present the changes as a series of coordinated steps, as this leads to a most straightforward mechanical understanding of the protein. The following is a description of the whole transition given along these lines. We begin with the change in α_γ_.

### Rearrangement of β-sheet in the α subunits

**Fig. 4:**
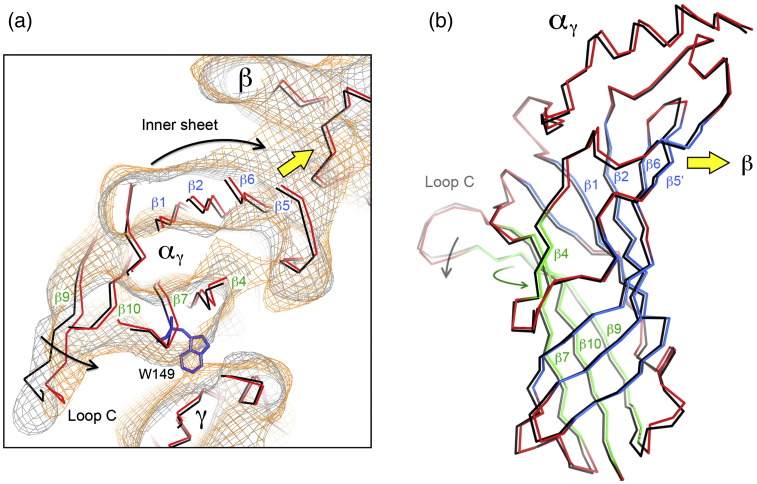
ACh binding to α_γ_ triggers a rearrangement of its inner and outer β‐sheets. (a) Slab through the closed- and open-class density maps at the level of the ACh binding site (grey contours, closed; orange contours, open), and the superimposed fitted C^α^ backbones (black, closed; red, open). The location of W149, a key ACh-binding residue, is also shown. Contours at 1 σ. (b). Whole extracellular portion of the α_γ_ subunit viewed from the lumen of the channel. The superimposed C^α^ backbones are coloured as in (a), but with the inner and outer sheets of the open channel coloured blue and green, respectively. Arrows in (a) and (b) denote ACh-induced displacements (see the text).

Figure 4a shows the superposed C^α^ backbones of the closed- and open-channel structures in the region of the α_γ_ binding site, after fitting the atomic model^5^ to the respective density maps (Methods). The two traces are displaced relative to each other, reflecting corresponding shifts in the density distributions. These displacements occur not only around loop C but also over the main body of the extracellular part of α_γ_. In the open-channel structure, the outer-sheet strands β9 and β10 (contiguous with loop C) are rotated towards a tryptophan, αW149,^34^ and other amino acids that coordinate with ACh^2,18^ on the other side of the binding pocket. In contrast, the inner-sheet strands β1, β2, β6 and β5′ are displaced in an approximately orthogonal direction, giving rise to a displacement of almost 1 Å at strand β5′, where α_γ_ touches the β subunit (yellow arrow, Fig. 4a).

The structural change indicated in Fig. 4a, although minor, is precisely defined by the ~ 6‐Å structures. If, for example, the images in each class (closed and open) are divided in two, and independent density maps are calculated from each half, the corresponding pairs of fitted β-sheets in the same class trace the same paths with small error [rmsd(C^α^_closed_) = 0.30 Å; rmsd(C^α^_open_) = 0.38 Å], and the differences between the two classes are qualitatively the same (Fig. S4).

Furthermore, separate motions of the whole inner and outer sheets of the β-sandwich are involved (Fig. 4b). When loop C is drawn in towards the binding pocket, it brings the outer sheet with it. There is an ‘in-plane’ rotational component, involving strands β9, β10, β7 and β4 (Fig. 4a; green arrow, Fig. 4b), which brings one edge of the β-sheet (strand β4) closer to the β subunit on the lumenal side of α_γ_, and a tilt component (grey arrow, Fig. 4b; see also Fig. 2b), which brings the other edge (strand β9) closer to the β subunit on the external side of α_γ_. The inner sheet is displaced toward the β subunit (yellow arrow, Fig. 4b) to accommodate these changes. The β-sheet rearrangement is therefore of a similar character, but smaller in magnitude than is needed to align the β-sheets of α in the closed channel with those of the non-α subunits or the protomer of AChBP.^17–19^ Quite possibly, these larger realignments reflect more nearly the rearrangement needed to achieve a desensitised state.

With the α_δ_ subunit, the changes around the ACh binding site are too small to allow a reliable description, but the inner sheets appear to be displaced sufficiently to affect the neighbouring γ subunit (Figs. S5 and S6). The smaller changes in α_δ_ would be consistent with electrophysiological and biochemical evidence that this subunit has a much higher closed-state affinity for ACh than α_γ_ in *Torpedo*,^35,36^ and so would make a lesser energetic contribution to the conformational change.

**Fig. 5:**
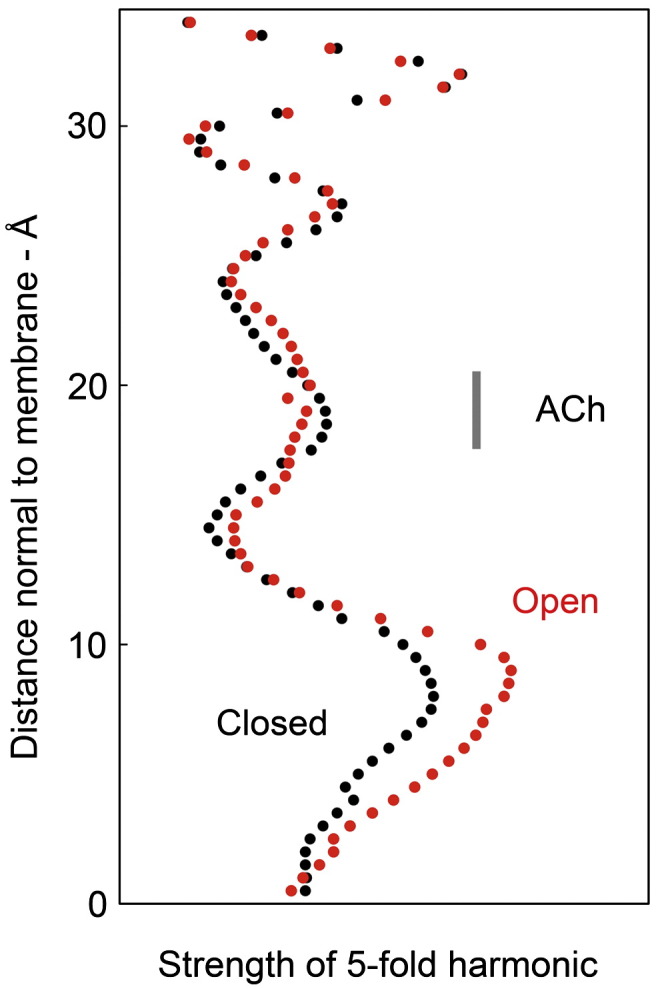
Variations in 5-fold strength at different levels through the ligand-binding domain. Power spectra were calculated from 1‐Å-spaced sections through the two density maps, and the relative strength of the 5-fold harmonic at successive sections through the structure is plotted (closed class, black; open class, red). The open channel displays stronger 5-fold symmetry, especially in the lower portion where the inner and outer β-sheets are most extensive and pack against each other. The location of ACh (based on the aligned structure of AChBP with bound carbamylcholine^18^) is indicated.

A principle of the allosteric mechanism, suggested by earlier studies,^5,19^ is that the two α subunits in the closed state are ‘distorted’ by their interactions with neighbouring subunits and that these distortions should lessen when ACh binds, making the α and non-α subunits structurally more alike. This principle is corroborated by the results. For example, the β-sheets of α_γ_ align more closely with those of γ after the rearrangement [rmsd(C^α^) = 1.39 Å] than before [rmsd(C^α^) = 1.46 Å], and similar differences are found in other comparisons. Most strikingly, the pseudo-5-fold symmetry of the ligand-binding domain becomes stronger in the open-channel form, indicating more regular packing of inner and outer β-sheets around the ring (Fig. 5).

### Displacement of non-α subunits in ligand-binding domain

We note that the extracellular part of the β subunit is displaced by an equal amount and in the same direction as the juxtaposed inner sheet of α_γ_ (yellow arrow, Fig. 4a). Likewise, the extracellular part of the γ subunit is displaced by an equal amount and in the same direction as the (geometrically equivalent) inner sheet of α_δ_ (Fig. S5). This suggests that the α subunits may propagate the conformational change in the ligand-binding domain by ‘pushing’ their inner sheets against their adjacent non-α neighbours. Indeed, comparison of equivalent cross sections through the two density maps shows that the extracellular parts of γ and β respond by moving roughly in the directions of their respective juxtaposed inner sheets, whereas the extracellular part of δ—which does not touch the inner sheet of either α subunit—moves the least.

**Fig. 6:**
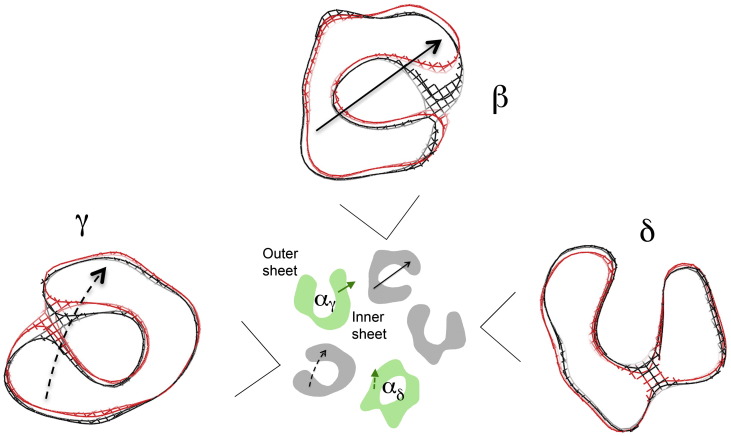
ACh-induced displacements of the non-α subunits at the base of the ligand-binding domain. Superimposed cross sections through the core β-sheet densities of β, γ and δ are shown, enlarged relative to the central pentamer, with arrows to indicate the displacements of β and γ. As indicated on the pentamer, these displacements, and those of the α inner sheets (green), all point in roughly the same direction (see also Fig. S6). Black and red are for the closed- and open-class maps; contours at 1.3 σ. ACh-induced displacements of the non-α subunits at the base of the ligand-binding domain. Superimposed cross sections through the core β-sheet densities of β, γ and δ are shown, enlarged relative to the central pentamer, with arrows to indicate the displacements of β and γ. As indicated on the pentamer, these displacements, and those of the α inner sheets (green), all point in roughly the same direction (see also Fig. S6). Black and red are for the closed- and open-class maps; contours at 1.3 σ.

Figure 6 depicts these movements of the non-α subunits at the base of the ligand-binding domain, where the pairs of the inner and outer β-sheets are most extensive and face one another, giving the core densities of each subunit a distinctive shape. As the figure indicates, β and γ appear to move as rigid units, since they display essentially the same cross sections in either map; yet, δ, to a close approximation, maintains a fixed position in the pentamer.

How then does the outward movement of β—the major disturbance detected by the *t* tests (Fig. 2)—originate? An obvious interpretation is that both α subunits and the intervening γ subunit participate actively in bringing it about. The displacements involved are all in roughly the same direction (Fig. 6; Fig. S6). Furthermore, the rmsds of the β-sheet backbones (pairwise comparisons of the closed- and open-channel inner sheets) increase in the sequence α_δ_ → γ → α_γ_ as 0.41 Å → 0.44 Å → 0.58 Å. The displacements are therefore coordinated around the pentamer. Whereas α_γ_ is mainly responsible for the movement of β, it appears that α_δ_ may contribute indirectly by using displacement of γ to propagate the α_δ_‐specific change. This set of interactions potentially explains how the two α subunits act concertedly to open the channel, despite no detectable cooperativity in ACh binding.^37^

### Communication to the membrane

The extracellular part of each subunit interacts, via connecting loops, with the ends of four α-helical segments and the linker M2–M3, protruding from the membrane surface. Most important are the Cys-loop joining the inner to the outer β-sheet, which overlies the helices M1, M3 and M4, and the tight β1/β2 loop of the inner sheet, which overlies the end of the pore-lining helix, M2.^4,5^ In addition, M1 is connected through the polypeptide chain. As might be expected, given the small magnitude of the displacements and the inherent flexibility of contacting loops, the movements of the extracellular parts of the subunits are not transferred directly to the membrane domain.

**Fig. 7:**
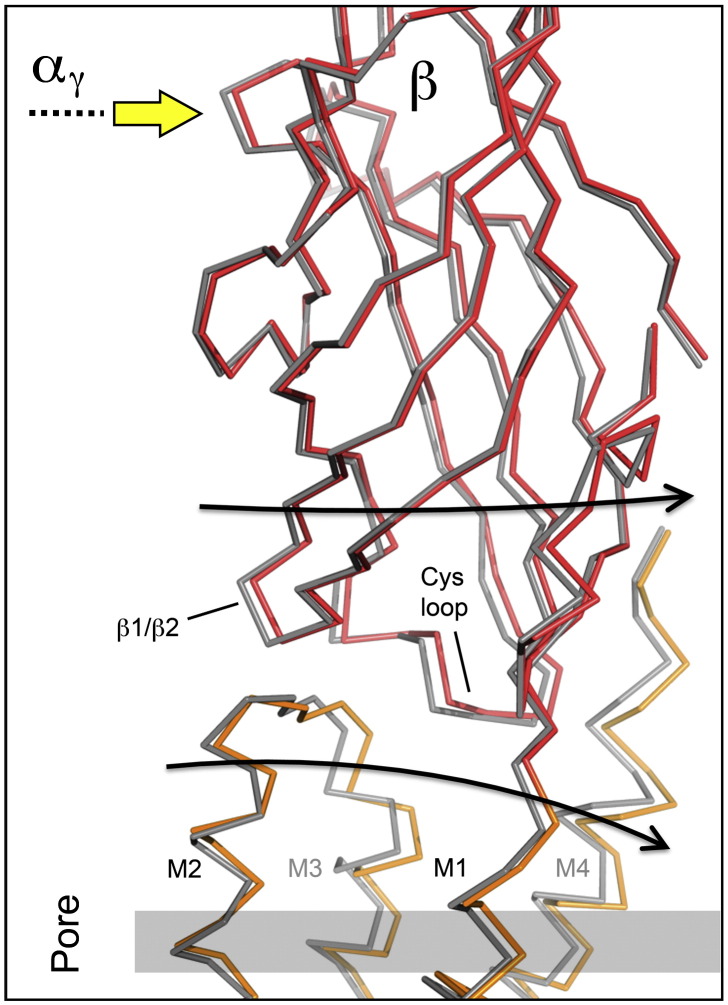
The outward displacement of β is transmitted to the membrane. Superposition of the fitted C^α^ backbones (closed channel, grey; open channel, red or orange) shows that the displacement of β, driven by α_γ_ (yellow arrow), involves tilting of the extracellular and membrane portions (curved arrows) about their shared the interface, located ~ 10 Å above the membrane surface (grey bar). Both parts move as rigid bodies (see also Figs. 6 and 9e and Supplementary Movie 1). The outward displacement of β is transmitted to the membrane. Superposition of the fitted C^α^ backbones (closed channel, grey; open channel, red or orange) shows that the displacement of β, driven by α_γ_ (yellow arrow), involves tilting of the extracellular and membrane portions (curved arrows) about their shared the interface, located ~ 10 Å above the membrane surface (grey bar). Both parts move as rigid bodies (see also Figs. 6 and 9e and Supplementary Movie 1).

In fact, β is the only subunit where displacement in the extracellular portion is tightly coupled, across the loop region, to equal displacement of the membrane helices (Fig. 7; see also Fig. 3d). In response to the change in α_γ_ (and the other subunits as described above), the extracellular part of β moves away from the axis of the receptor, tilting slightly to maximise displacement near the membrane surface. The underlying set of four helices tilt by ~ 2° in the same direction, but in the opposite sense, to match this displacement (Fig. 7). In other words, the β subunit has two conformations that are related by a slight rocking of the extracellular and membrane components about their shared interface (Supplementary Movie 1). This motion of the β subunit must be central to the gating mechanism since it is the only resolvable change across the two domains, which communicates the effect of ACh binding to the membrane and hence to the gate. ABC transporters appear to use a similar kind of motion to translocate substrates across membranes in response to ATP.^38^

### Membrane helices

The helical segments of the five subunits arrange regularly in the membrane, forming concentric rings around the pore. In the closed-channel structure, the M2 helices composing the inner ring bend inward and come together near the middle of the membrane (Fig. 3b), creating a hydrophobic constriction, believed to function as the gate of the channel.^3,4,7,39,40^ Helices in the next ring, M1 and M3, pack more tightly side to side, creating a wall that separates the M2s from the lipids. The M4 helices of the outermost ring make limited contact with the rest of the protein and are not as well defined in the density maps, suggesting that they are less rigidly held than the others.

Differences between equivalent helices in the closed- and open-class maps are greatest in the extracellular leaflet of the lipid bilayer. At this level (dashed bar in Fig. 3b), although most helices are in matching locations (Fig. 3d), some clearly are displaced relative to each other. First, all four open-class helices of the β subunit lie slightly further from the pore axis (broken lines and arrow, Fig. 3d), due to their ~ 2° outward tilt. Second, the open-class M2 helix of α_γ_ is shifted by about 1 Å toward α_γ_M3. Third, the open-class M2 helix of δ is shifted by about 2 Å in the same direction as the helices of the β subunit.

**Fig. 8:**
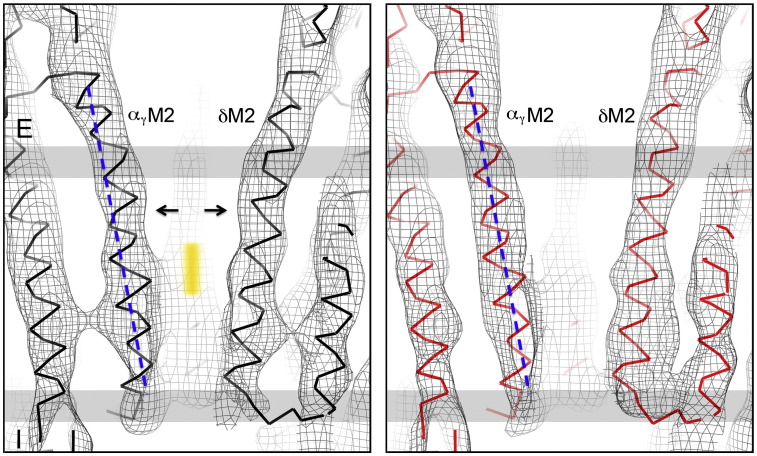
Flexure of pore-lining helices contributes to widening of the pore. Shown are closed- and open-channel densities (left and right panels, respectively), with superimposed C^α^ backbones, in a near-axial slab containing α_γ_M2 and δM2. These helices increase the dimensions of the pore by straightening (arrows) in response to ACh binding, as indicated for α_γ_M2 by comparison with the overlaid broken line. The straightening of δM2 is less obvious in this figure because it is predominantly tangential to the axis of the pore (see Fig. 9d). Vertical orange bar identifies the assumed location of gate. Membrane, grey bars; E, extracellular; I, intracellular. Contours at 1 σ.

Views normal to the channel axis, with the fitted C^α^ backbones (Fig. 8), show that the shifts of α_γ_M2 and δM2, in cross section, are due mainly to their different curvatures in the two maps. In the closed-channel configuration, α_γ_M2 bows inward toward the gate (orange bar), whereas in the open-channel configuration, it follows a straight line (indicated by the broken line). The δM2 helix also bows slightly inward toward the gate in the closed-channel configuration but straightens predominantly in a tangential direction when the channel opens (see below). The α_γ_M2 and δM2 helices therefore flex between alternative bent and straight conformations (Supplementary Movie 2).

**Fig. 9:**
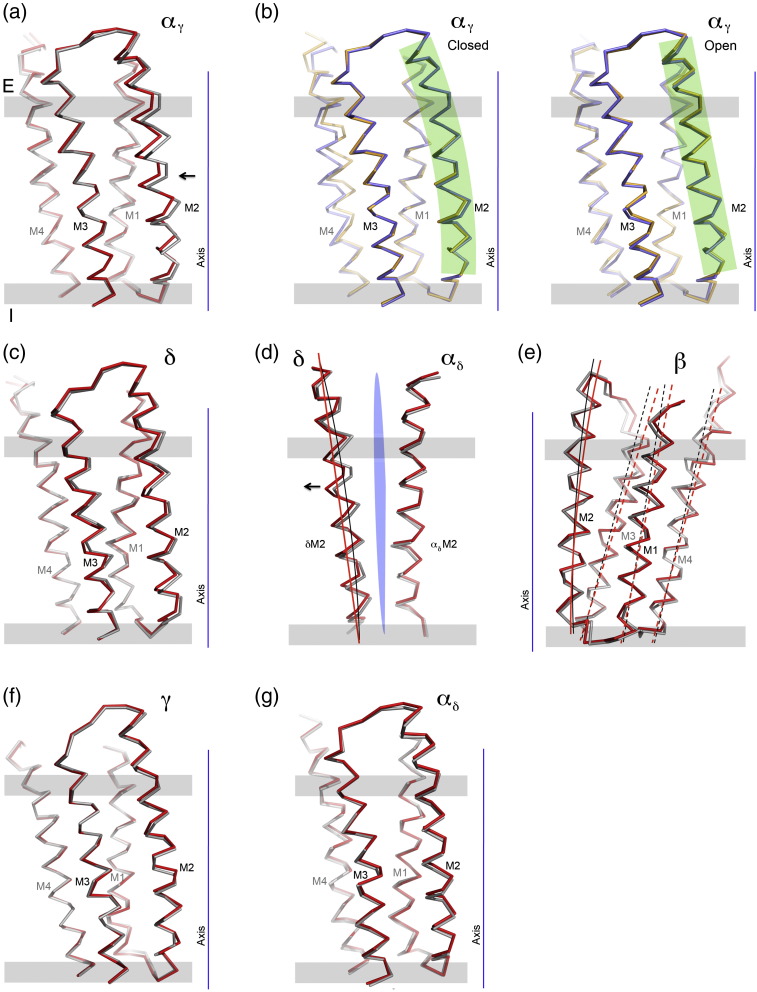
The principal gating movements are mediated by the membrane helices of α_γ_, δ and β. (a) Superposition of the C^α^ backbones of the α_γ_ helices in the closed (grey) and open (red) channel structures, showing that α_γ_M2 straightens when the channel opens. (b) Superposition of α_γ_M2 helices (orange and blue traces) in the closed (left) and open (right) class, determined from independent half data sets; green shading highlights the alternative curved and straight conformations of α_γ_M2. (c) Superposition of the δ helices in closed- and open-channel structures. Flexure of M2 in the δ subunit is accompanied by a similar (but lesser) flexure of M3, whereas the other helices (M1 and M4) do not change appreciably. (d) Crevice (blue wedge) exposed between δM2 and α_δ_M2 by straightening of δM2, as indicated by a pair of overlaid lines (viewed from inside the pore; see also Fig. S7). (e) Tilting of membrane helices in the β subunit. All fourhelices of β tilt by ~ 2° to open the channel, as indicated by the pairs of overlaid lines. (f and g) Superposition of membrane helices in γ and α_δ_: the helices in these subunits have nearly identical closed and open conformations (see also Fig. S8). The closed-channel (grey) and open-channel (red) colour scheme is used throughout; arrows indicate directions of main displacements. Membrane, grey bars; E, extracellular; I, intracellular. The principal gating movements are mediated by the membrane helices of α_γ_, δ and β. (a) Superposition of the C^α^ backbones of the α_γ_ helices in the closed (grey) and open (red) channel structures, showing that α_γ_M2 straightens when the channel opens. (b) Superposition of α_γ_M2 helices (orange and blue traces) in the closed (left) and open (right) class, determined from independent half data sets; green shading highlights the alternative curved and straight conformations of α_γ_M2. (c) Superposition of the δ helices in closed- and open-channel structures. Flexure of M2 in the δ subunit is accompanied by a similar (but lesser) flexure of M3, whereas the other helices (M1 and M4) do not change appreciably. (d) Crevice (blue wedge) exposed between δM2 and α_δ_M2 by straightening of δM2, as indicated by a pair of overlaid lines (viewed from inside the pore; see also Fig. S7). (e) Tilting of membrane helices in the β subunit. All four helices of β tilt by ~ 2° to open the channel, as indicated by the pairs of overlaid lines. (f and g) Superposition of membrane helices in γ and α_δ_: the helices in these subunits have nearly identical closed and open conformations (see also Fig. S8). The closed-channel (grey) and open-channel (red) colour scheme is used throughout; arrows indicate directions of main displacements. Membrane, grey bars; E, extracellular; I, intracellular.

As with the β-sheet rearrangement (Fig. 4), the conformations and relative positions of individual helices are precisely defined by the ~ 6‐Å structures, allowing interpretation of small changes that can be validated using independent half data sets (Methods). Consider, for example, the helices of α_γ_ in the superimposed closed- and open-channel configurations (Fig. 9a). We observe that the straightening of α_γ_M2 produces a maximum displacement of 1.5 Å (arrow, Fig. 9a) away from the axis of the pore in the direction of α_γ_M3; yet, there is no appreciable movement of M1 or M3. In fact, the same two configurations of M1, M2 and M3 are accurately reproduced in independent structures determined from only half the number of images (Fig. 9b). The observations based on the full data sets are therefore genuine and not influenced significantly by errors due to over-fitting or by inadequate quality of the density maps. We infer that α_γ_M2 moves independently of the other helices making use of available interstitial space.^4^

The straightening of δM2, while similar to that of α_γ_M2, is not equivalent since it is accompanied by slight straightening of δM3 (Fig. 9c). Also, the direction of maximum flexure of δM2 is near-tangential to the channel axis (arrow, Fig. 9d), rather than radial as is the case with α_γ_M2 (see Fig. S7). As a result, a 1‐ to 2‐Å‐wide crevice is created between the pore-lining helices δM2 and α_δ_M2 (blue wedge, Fig. 9d), eliminating the tight side-to-side interaction that may be critical in stabilising the hydrophobic gate.^4^ Of the other M2 helices, βM2 tilts together with βM1, βM3 and βM4 (Fig. 9e), and γM2 and α_δ_M2 have a similar conformation in either structure (Fig. 9f and g). Interestingly, α_δ_M2 does not achieve a bent conformation, like α_γ_M2, when the channel is closed (Fig. S8).

In summary, the β subunit undergoes the greatest overall displacements both in the membrane and in the ligand-binding domains, and so is the primary component communicating the effect of ligand binding to the helices around the pore. Tilting of the pore-lining helix βM2 is accompanied by straightening of the neighbouring helices, α_γ_M2 and δM2. By flexing radially and tangentially, these two helices modulate the shape, dimensions and polarity of the pore.

### Wider pore

The diameter of the closed pore is about 6 Å at the assumed level of the gate (orange bar, Fig. 8), where the lumen is both constricted and completely encircled by hydrophobic side chains.^4^ This region and the nearby extracellular region form an energy barrier to ion permeation across the membrane because they contain no polar groups to substitute for the hydration shell and so the ion is, in effect, too big to pass readily through.^4,39,40^ In contrast, the open pore contains no such barrier and the narrowest part, according to mutation combined with electrophysiological studies,^41,42^ is near the intracellular membrane surface, where polar residues are available to keep the ions solvated.

**Fig. 10:**
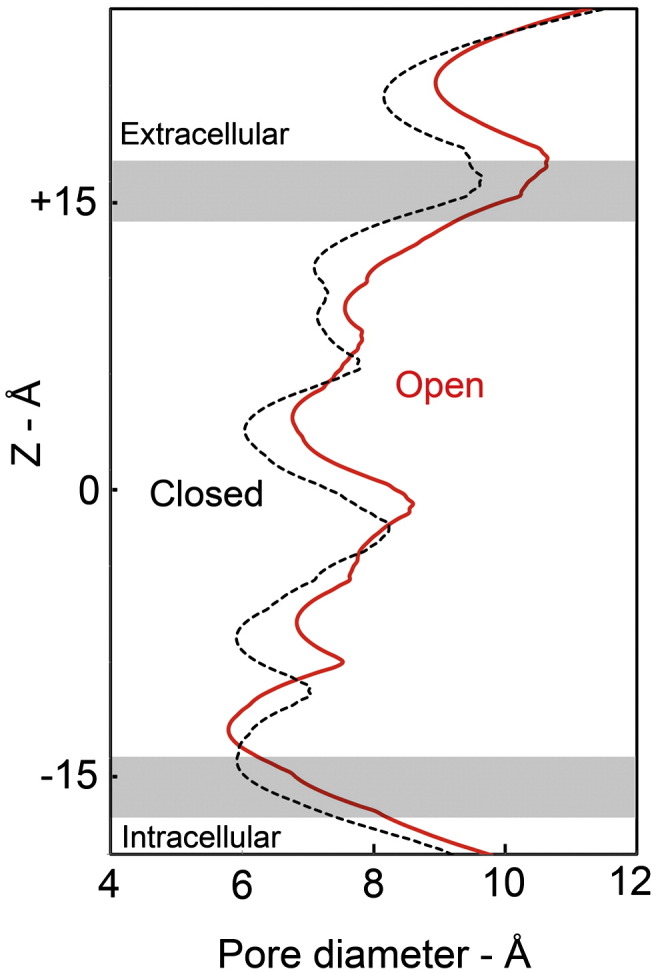
ACh-induced change to the limiting diameter of the pore, calculated with the program HOLE,^43^ after fitting the coordinates of the atomic model^5^ (PDB ID: 2BG9) to the closed- and open-class density maps (black and red curves, respectively). The diameter of the pore increases in the constricting hydrophobic region near the middle of the membrane (at *Z* = 0), and the narrowest part shifts to the intracellular membrane surface where the pore is lined by polar residues. (The constricting hydrophobic region can be identified with the bulge on δM2 at the level of the orange bar in Fig. 8).

As Fig. 9a–e shows, the asymmetric helix movements triggered by ACh binding perturb the pore most at the level of the gate and in the nearby extracellular region. These movements must lower the energy barrier for ion permeation in several ways. First, they increase the limiting diameter of the pore (as sensed by a sphere) near the middle of the membrane by about 1 Å (Fig. 10). This shifts the narrowest part to the intracellular membrane surface, in agreement with the electrophysiological results. Second, they increase the cross section of the pore unevenly, making room inside the pore for additional water molecules. Third, they diminish the hydrophobicity of the pore lining by introducing crevices (as in Fig. 9d), which will expose buried polar groups.

Are these changes in the dimensions and chemistry of the pore lining sufficient to achieve a fully conducting open state? Experimental limitations such as the presence of closed channels in the open-class images, and imperfect sorting of the images into the two classes, could have led to an observed change that is less than the actual physiological one. On the other hand, the apparently complete unbending of pore-lining helices (Fig. 9b and d) seems likely to mark a transition endpoint. The fact that the open α_γ_M2 conformation matches, and is not in-between, the (fixed) conformation of α_δ_M2 (Fig. S8) reinforces this argument. We suggest therefore that the observed movements reflect very nearly the actual extent attained *in vivo*. This would be consistent with molecular dynamics simulations based on the closed-channel structure,^44–47^ as well as measurements made on the functioning protein,^12^ indicating that only minimal adjustments to the pore are needed to obtain the measured conductance of the open-channel form.

## Discussion

The spray-freeze-trapping experiments reported here have allowed a detailed description of the rapid gating movement of the fully intact ACh receptor under conditions that recapitulate the brief activation event at the nerve–muscle synapse. This description illuminates the underlying allosteric mechanism and reveals that gating is an asymmetric process, correcting a preliminary lower-resolution study where the membrane domain was assumed to be 5-fold symmetric.^8^ We discuss below these basic properties of the heteropentamer.

### Asymmetric gating

The near-perfect 5-fold arrangement of M2 helices encircling the closed pore (indicated by the pentagonal broken lines in Fig. 3d) contrasts with the asymmetric arrangement of M2 helices encircling the open pore and makes distinct the two configurations of pore-lining helices. The symmetry of the closed pore probably arises because the M2 helices bending inward to form the gate are somewhat separated from the outer protein wall and held together by a small number of side-to-side contacts involving the same highly conserved amino acid side chains.^4^ We have proposed that this organisation makes the assembly of M2s intrinsically unstable, since its integrity would depend on equal sets of interactions between each of its components.^4^ When a disturbance is introduced (as with βM2 tilting out of the ring), at least one set of interactions is eliminated. The whole assembly should then break up, with the remaining helices now freed to adopt the configuration they would have if these interactions did not exist. The asymmetric appearance of the open pore is explained simply on this basis, since the interstitial space available to the freed helices would vary from one subunit to the next (see Supplementary Movie 3).

**Fig. 11:**
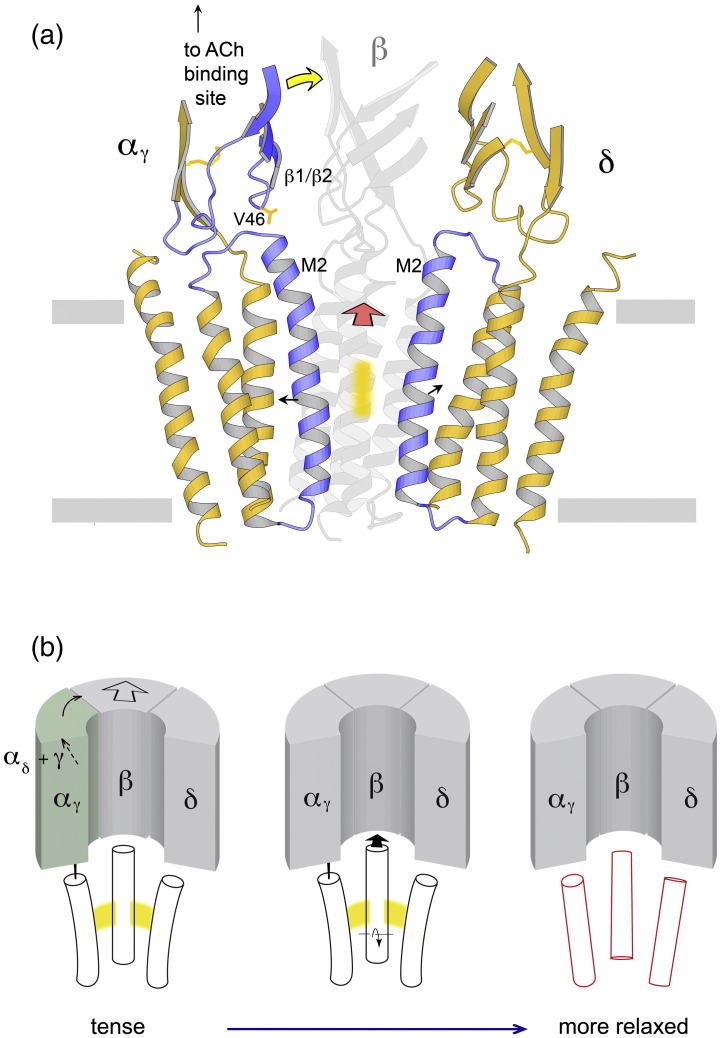
Structural mechanism of gating. (a) Proposed principal gating action: binding of ACh induces displacement of the inner β-sheet of α_γ_ (yellow arrow), which pushes out β (red arrow); this destabilises the helical arrangement forming a central hydrophobic gate (orange bar), releasing the bent α_γ_M2 and δΜ2 helices (short arrows) that can now straighten, opening up the pore. Ribbon diagram is for the closed channel.^5^ Main moving parts of α_γ_ and δ in blue. (b) Overall allosteric model: ACh binding relieves ‘distortions’ in the α subunits, causing displacements that combine (thin arrows, left) to push the extracellular part of β outward (thick arrow, left). The outward movement of β propagates into the membrane (arrows, middle), breaking symmetrical interactions between pore-lining helices (yellow), allowing them to adopt an alternative ‘freed’ configuration (right) that is permeable to ions.

The pore-lining helix βM2 and its immediate neighbours, α_γ_M2 and δM2, are the principal elements responsible for the widening and reduction in pentagonal symmetry of the pore that occurs when it opens. Figure 11a shows these helices in relation to the gate region of the closed channel and to contacting loops of the ligand-binding domain, in particular the loop β1/β2 on the inner sheet of α_γ_. This loop interacts through V46 with the end of α_γ_M2^4,11,48^ and may affect the relative stabilities of its two helix conformations, and hence the gating equilibrium,^48^ as a result of disparate motions between the two domains. We suggest that α_γ_ is the primary subunit determining initiation of an open event, using the displacement of its inner sheet (yellow arrow, Fig. 11a) to push β outward (red arrow), thereby controlling flexure of α_γ_M2 and δM2 (small arrows). On the other hand, δ may influence the duration of an open event, since δM2 is the most ‘misaligned’ helix in the open configuration (Fig. 3d) and requires the greatest readjustment to reassemble the symmetrical closed-pore ring.

### Allosteric mechanism

The pseudo-5-fold symmetry of the receptor changes as a result of the overall conformational change, becoming stronger in the lower part of the ligand-binding domain, where the inner and outer β-sheets face each other (Fig. 5), but weaker in the membrane (Fig. 3d). These changes support and extend an earlier model for the allosteric mechanism, which was restricted to the ligand-binding domain.^5,19^ In terms of the whole structure, this model is as follows (Fig. 11b). The two α subunits in the closed state have ‘distorted’ arrangements of β-sheets. Coordination of ACh with amino acids in the binding pockets (partially) overcomes these distortions, allowing an arrangement closer to that of the non-α subunits and thus promoting an increase in 5-fold symmetry. The associated movements in the α subunits combine, by transmission through the intervening (γ) subunit, to push β outward (Fig. 11b, left). Propagation of the outward displacement into the membrane (Fig. 11b, middle) breaks the symmetrical side-to-side interactions between pore-lining helices, and they adopt energetically more favoured shapes (but a less symmetrical configuration; Fig. 11b, right). Hence, in both domains, the structures shift from a tense toward a relaxed state.

This mechanism of rapid switching between two pore configurations provides a physical basis for explaining the intricate gating patterns observed in single-channel recordings. Early electrophysiological experiments on calf–*Torpedo* hybrid receptors highlighted the importance of the α and δ subunits in determining duration of channel opening.^49^ Our results show how α_γ_ and δ may strongly influence breakup and reassembly of the gate-forming ring. The α_γ_ and δ subunits are also implicated in several well-characterised mutations causing congenital myasthenic syndrome (CMS) in humans. For example, abnormally brief channel openings occur with the CMS mutation αV285I,^50^ and large increases in channel open time occur with the CMS mutation δS268F.^51^ Since both amino acids project into the interstitial space behind α_γ_M2 and δM2, these mutations would affect directly the relative stability of the bent (closed) and straight (open) helix conformations.

In the present study, the limited resolution has precluded a description of the channel-opening mechanism in detailed chemical terms. Nevertheless, our findings put into a structural context the results of some recent biophysical studies probing aspects of muscle-type ACh receptor gating.^11,12,52–55^ For example, the rearrangements in the α subunits, to initiate the conformational change, would be facilitated by loose packing of the inner and outer sheets due to polar amino acids buried in the β-sandwich core.^54,56^ The small helix motions to open the channel, inferred by proton transfer measurements,^12^ are directly apparent. Can further insight now be obtained from comparison with the higher‐resolution x-ray structures of related homomeric channels? This would not be straightforward because the simpler ion channels would be unable to incorporate the diversity of movements that characterises the ACh receptor. On the other hand, it seems quite likely that other heteromeric members of the Cys-loop family may operate by principles similar to those we have described here.

## Conclusion

The closed-to-open transition of the ACh receptor is a coupled process linking two domains that are not only structurally, but mechanistically distinct. The ligand-binding domain, through the concerted action of several subunits, magnifies and focuses the ACh-triggered displacements into a single subunit. The membrane domain has an architecture that lends a specific kind of instability to the pore-lining elements, where the effect of a single perturbation is amplified by the symmetry present, causing their arrangement to flip. The complete conformational change can be understood in terms of an allosteric mechanism in which the binding of agonist relieves preexisting tense structures in both domains.

The gating movement described here is central to fast neuromuscular transmission in all vertebrates and would have been fine-tuned through evolution to optimise the speed of initiation and termination of the postsynaptic response. In the ligand-binding domain, the relative displacements are too small to necessitate significant readjustment of non-covalent bonds, which may slow down a conformational change. In the membrane domain, the key to rapid gating movement may be the special architecture that enables the pore-lining helices, mainly by flexing, to alternate the local geometry and chemistry of the pore.

## Methods

### Specimen preparation

Tubular ACh-receptor crystals were grown from isolated *Torpedo* postsynaptic membranes in 100 mM sodium cacodylate; 1 mM calcium chloride, pH 7.0.^57^ The spray-freeze-trapping experiments used tubes belonging to the (− 15,5) and (− 17,5) helical families.^22^ An additional analysis of the receptor in the closed-channel form used untreated tubes belonging to the (− 16,6) and (− 18,6) helical families.^29^ Spraying and freeze-trapping of samples on the electron microscope grids^20^ were performed at 8 °C and 80–90% relative humidity. The spray solution was 100 mM ACh chloride and 1 mg ml^−^ ^1^ ferritin, pH 7.0.

### Electron microscopy

Tubes in the frozen films were imaged at 4K (magnification, 38,500 ×; defocus range, 1–2 μm; electron dose, 25 electrons/Å^2^) over holes in pre-irradiated carbon support film.^29^ The instrument was a JEM-3000FS field emission electron microscope operating at 300 kV and incorporating a liquid-helium‐cooled top-entry stage.^24^

### Data processing

Electron micrographs were screened initially by optical diffraction to eliminate tubes showing obvious disorder. Selected tubes, giving symmetrical diffraction patterns with layer lines visible to 35 Å resolution, were digitised at 1.3 Å equivalent pixel dimension using a Joyce-Loebl Mark IIIc microdensitometer, modified extensively in-house. Distortion correction was performed on each image using a segmental Fourier–Bessel approach,^58^ with 50% overlap between successive segments. The template for aligning each segment was an averaged set of layer lines from previously analysed images in the relevant helical family, cut to a resolution of 20 Å. No prior selection was made according to whether the image was determined later to belong to the closed or open class. Each corrected image was evaluated in Fourier space for possible inclusion into data sets by analysing the quality of radial 2-fold symmetry and retention of signal at high resolution (based on nearness to ideal phases of 0° or 180°). The use of 50% overlapping segments, rather than of adjacent segments in the distortion corrections,^58^ led to consistent improvements based on these criteria.

The two reference maps for the sorting procedure were calculated by averaging density maps reconstructed from single images: Ref(− ACh) from unsprayed grids and Ref(+ ACh) from sprayed grids where ferritin was also visible. Sorting of the ‘unlabelled’ images into closed and open classes was done by comparing equivalent sections in three-dimensional maps reconstructed from each image with either reference map and determining differences in correlation coefficient (Fig. 1b). Optimum discrimination was achieved by confining the comparisons to sections within the central two-thirds of the receptor and limiting the resolution to 20 Å.

To check the validity of this sorting method, which depends on small differences between correlation coefficients, we determined the significance of differences between the corresponding structures by calculating statistical *t* maps (Fig. S1; Fig. 2). Three-dimensional arrays of the mean densities and their standard deviations were calculated for each pair of structures by averaging (in real space) the relevant single-image maps. The differences between the mean densities at equivalent array points were then taken and used, in conjunction with the standard deviations, to generate an array of *t*, or significance values, which could be contoured and interpreted with reference to a Student's *t* table.

The final ~ 6‐Å maps were synthesised after combining the contrast-transfer-function-corrected Fourier terms from all images composing each data set in each helical family and real-space averaging the resultant pairs of maps.^29^ Effects of disorder were compensated by weighting down the low‐resolution (< 1/15 Å^−^ ^1^) terms.^4^ A negative temperature factor of − 150 Å^2^ was applied to correct for falloff in signal strength along the scanning direction of microdensitometer. The map for comparison from untreated tubes was determined using identical procedures (see Table 1). The estimated positions of the phospholipid headgroups (Fig. 3b) correspond to peaks in the densities calculated by Fourier inversion of terms along the equatorial layer line. The standard Fourier cutoff used for all three maps of 6 Å was changed to 10 Å in Fig. 6 in order to reduce side-chain contributions and emphasise the β-sheet cores of the subunits.

FSC and measurement of 5-fold strength were used to evaluate the resolution and quality of the final density maps. In the closed- and open-class maps, matching properties of the two FSCs (Fig. S3) showed that the resolution was not limited by short-range disorder due to ACh-induced conformational heterogeneity or due to the loss of a crystal contact at the radial 2-fold axis upon ACh binding (Fig. 2). A clear increase in 5-fold strength was observed at many levels in the open-channel map (Fig. 5), ruling out the possibility that shearing forces developed during spray impact, or some unsuspected factor, caused a systematic perturbation that might have biased the appearance of that map.

### Model building

Models for the open- and closed-channel conformations were refined by maximising the correlation between the experimental densities and the densities computed from an atomic model of the closed channel^5^ [Protein Data Bank (PDB) ID: 2BG9], using a deformable elastic network algorithm, with the program DireX.^59^ Strong, relatively rigid, deformable elastic network restraints were applied to obtain best fits during the refinement, while at the same time retaining about 85% of the amino acid residues in α-helices and in β-sheets with their original secondary structure. To evaluate the significance of small differences revealed between the two final closed- and open-class structures, we applied the same refinement procedures to independent density maps calculated from half data sets (i.e., data sets derived from the processed images after dividing them randomly into two equal halves). If the differences present in the structures derived from the full data sets were reproduced qualitatively in structures derived from the half data sets, they were considered significant: that is, genuine differences brought about by ACh binding and not a result of over-fitting to noisy data. All the described subunit and secondary structure displacements, as well as changes in helix conformation, were validated in this way. The refinements were not used to make deductions about loop regions, which are less reliably modeled, or about the absolute positioning of the polypeptide chains, which is limited by the accuracy of the original atomic model. In error and displacement calculations, and in Fig. 4b, the amino-acid-sequence-based assignments for β-sheet were as in Ref. 54. Calculations using the program HOLE^43^ (Fig. 10) assume knowledge of exact side‐chain positions and do not yield accurate absolute values, because an atomic structure of the ACh receptor is not yet available. Molecular drawings were prepared with UCFS Chimera^60^ (Fig. 3b) and PyMOL.^61^

### Accession numbers

The three-dimensional density maps have been deposited in the Electron Microscopy Data Bank under accession numbers EMD-2071 (closed class) and EMD-2072 (open class). The atomic models have been deposited in the PDB as 4AQ5 (closed class) and 4AQ9 (open class).

The following are the supplementary data related to this article.

Supplementary Movie 1

Supplementary Movie 2

Supplementary Movie 3

## Figures and Tables

**Table 1:** Image data and processing statistics

	Untreated tubes	Closed class		Open class	
	(No ACh)	Ref(− ACh)	Spray	Ref(+ ACh)	Spray
Helical families	(− 16,6), (− 18,6)	(− 15,5)	(− 15,5), (− 17,5)	(− 15,5)	(− 15,5), (− 17,5)
No. of images	72, 24	28	55, 28	28	67, 28
Tube diameters (Å)	770, 830	710	710, 760	710	710, 760
Molecules averaged	2.4 × 10^5^	5.9 × 10^4^	1.8 × 10^5^	6.7 × 10^4^	2.0 × 10^5^
Mean defocus (μm ± SD)	1.31 ± 0.24	1.46 ± 0.22	1.38 ± 0.24	1.43 ± 0.21	1.42 ± 0.21
Segment length (Å ± SD)^a^	602 ± 29	602 ± 29	597 ± 30	598 ± 31	591 ± 30
Resolution (Å)^b^	6.5	6.2		6.2	

a Length along tube used in distortion correction.

b Resolution estimated by FSC_0.5_ criterion (Fig. S3).
