# Supplementary material for: Gating Movement of Acetylcholine Receptor Caught by Plunge-Freezing
Source: J Mol Biol. 2012 Oct 5;422(5):617–34. doi: 10.1016/j.jmb.2012.07.010 (PMC3443390; doi:10.1016/j.jmb.2012.07.010)
Supplement: Supplementary file 1 — Supplementary Movie 1 [file mmc1.docx]

**Unwin and Fujiyoshi, 2012 Supplemental Data**


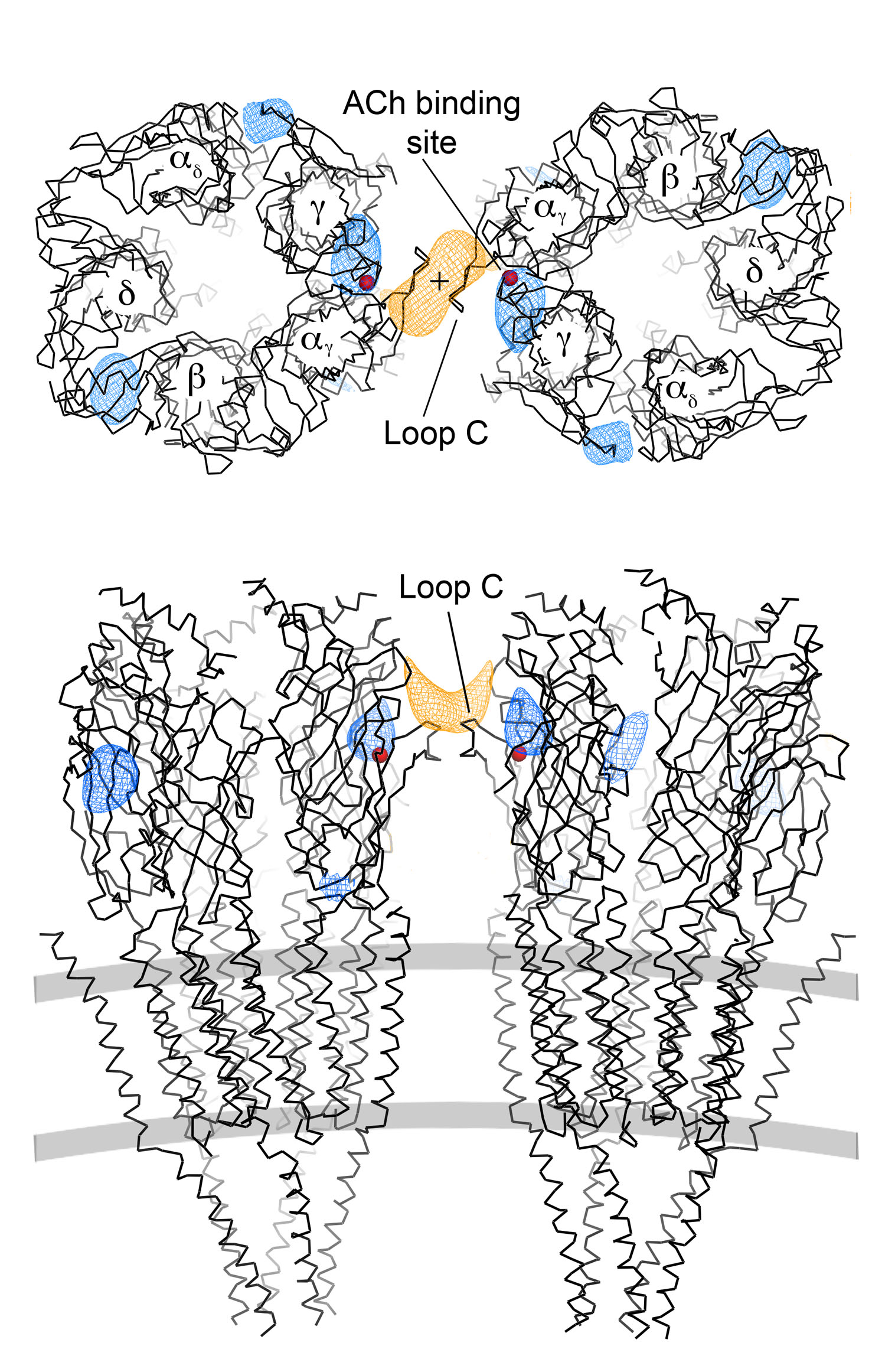


**(a)**

**(b)**

**Figure S1.** Statistical *t*-map showing the regions of most significant difference between the density maps, Ref(+ACh) and Ref (-ACh), where the images are from acetylcholine-exposed, ferritin-labelled tubes and untreated tubes, respectively. The map is superimposed on a pair of neighbouring receptors docked into the tube surface lattice. Views are (a) from the synaptic cleft and (b) parallel to the membrane. Red spheres: acetylcholine binding site in α_γ_. Contours at *P*=0.01: orange, decrease, blue increase in density on exposure to acetylcholine.

**Unwin and Fujiyoshi, 2012 Supplemental Data**


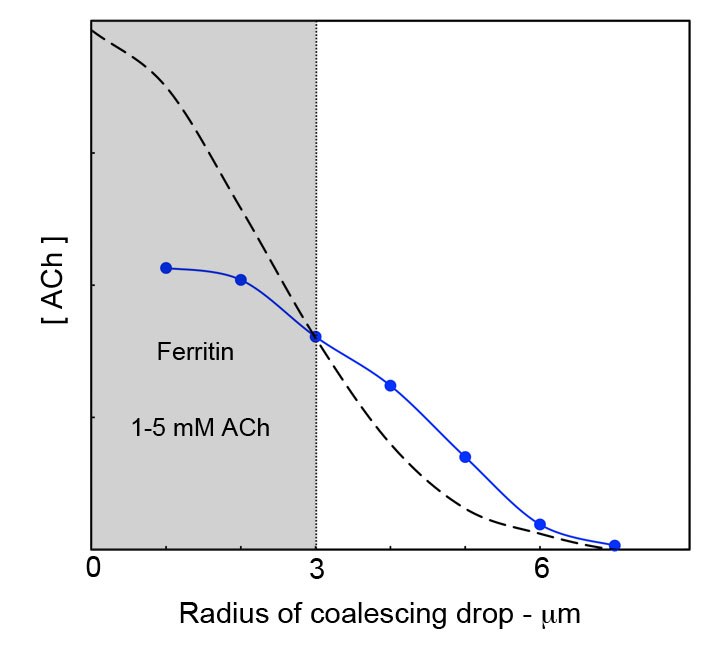


**Figure S2.** Rough estimate of extent of diffusion of acetylcholine ahead of a 1μm diameter droplet spreading on a thin aqueous film. Under the standard 10ms delay following spray impact the zone of coalescence (shaded area) would have attained a radius of ~3μm ^S1^, and acetylcholine would have spread a further ~3μm by diffusion (based on measurements using sodium ions^S1^, which have the same diffusion coefficient as acetylcholine: 1-1.3 x 10^-5^ cm^2^/sec^S2,S3^). The calculated [ACh] distribution from a disk source^S4^ best matches the experimental result (blue curve) after a diffusion time of 2ms (dashed curve). Thus 2ms is roughly the maximum duration unlabelled tubes (i.e. those lying outside the ferritin-delineated zone of coalescence) could have been exposed to significant concentrations of acetylcholine.

**Unwin and Fujiyoshi, 2012 Supplemental Data**

**
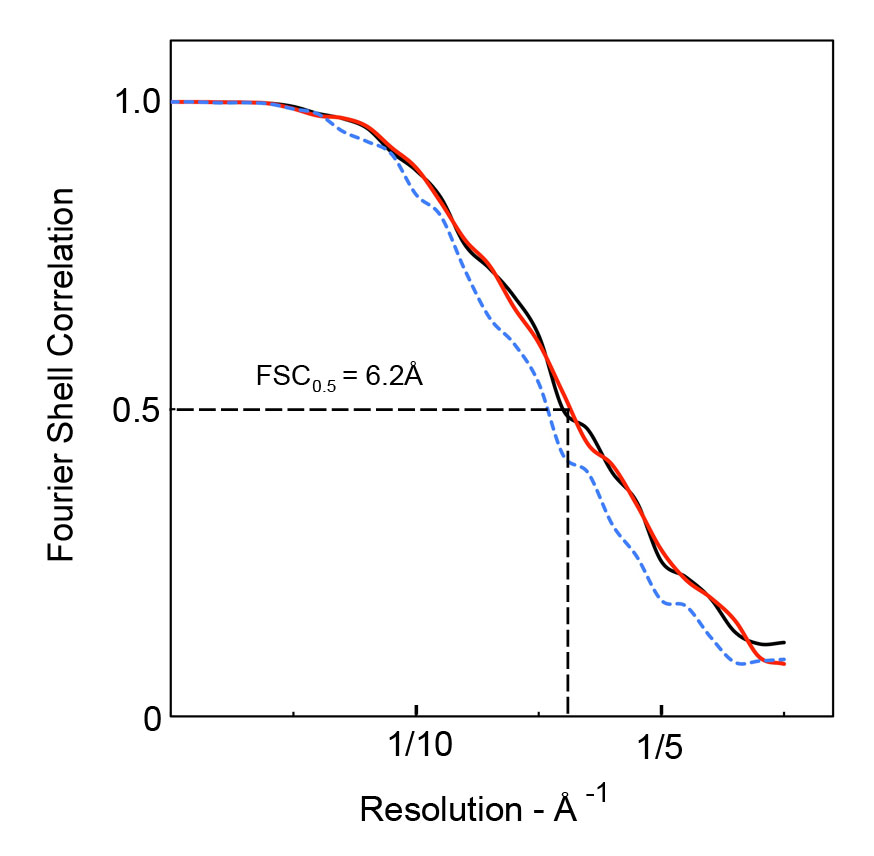
**

**Figure S3.** Fourier shell correlations to estimate the resolution of the closed- and open-class density maps, and also the map determined from untreated tubes (see Table 1). The data sets for each structure were split into independent half-sets, and maps were calculated from each half-set. Plotted are the cross-correlation coefficients between the pairs of maps over corresponding shells in Fourier space; black: closed class, red: open class; blue: untreated tubes. The resolution by the FSC_0.5_ criterion^S5^ is 6.2Å (vertical dashed line) for both the closed- and open-class maps, and 6.5Å for the map from untreated tubes.

**Unwin and Fujiyoshi, 2012 Supplemental Data**


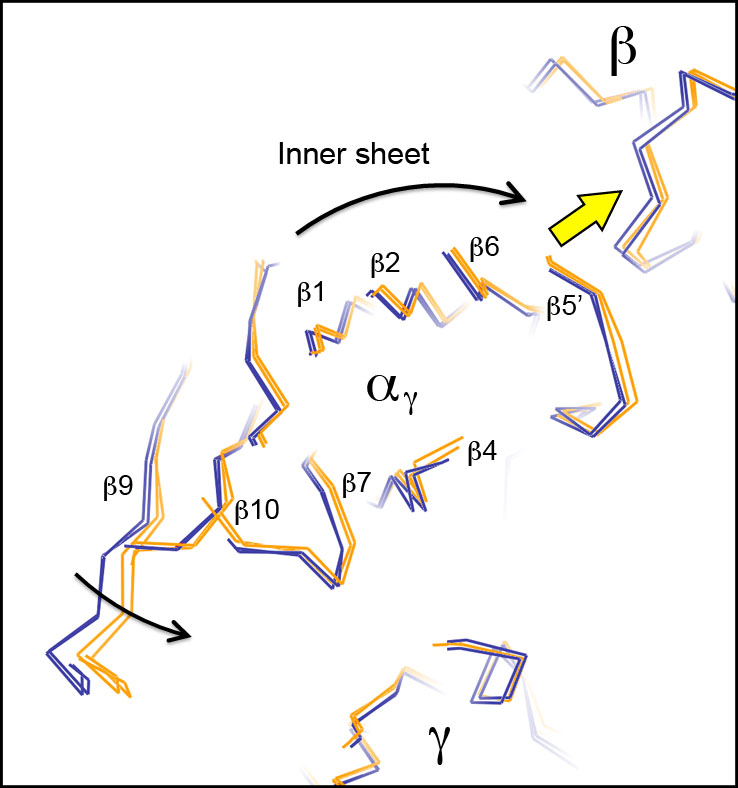


**Figure S4.** Validation of β-sheet rearrangement in α_γ_. The slab shown is the same as that in Fig. 4a, but in this example the superimposed backbones were determined by fitting the atomic model (PDB ID: **2BG9**) to the densities calculated from independent half-sets of data (see Methods). In either class, the pair of traces match one another with small error (blue traces: closed class; orange traces: open class) and the distinction between the two classes is clearly retained. This demonstrates that Fig. 4 gives an accurate representation of the displacements induced by acetylcholine, even though they are small.

**Unwin and Fujiyoshi, 2012 Supplemental Data**

**
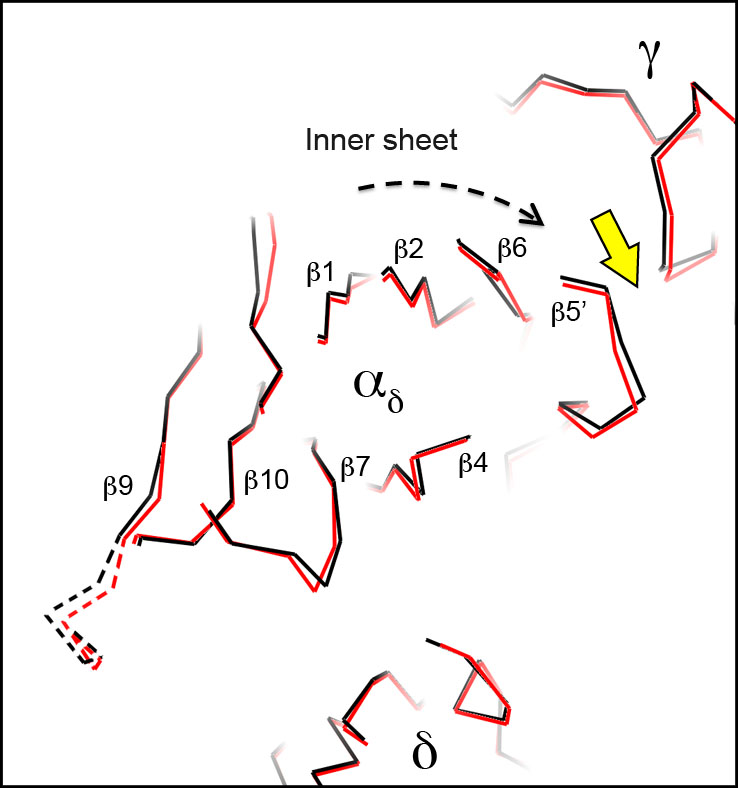
**

**Figure S5.** The β-sheet rearrangement in α_δ_ is smaller than in α_γ_. The slab shown through α_δ_, (and neighbouring γ and δ) is equivalent to that in Figs. 4a and S4. In this case, the changes in Cα backbones (black, closed; red, open) tracing loop C (which is not clearly resolved in either density map) and the outer β-sheets are too small to allow a reliable description. However there are small displacements of the inner β-sheets (curved arrow). The displacements of the inner sheets in α_δ_ are accompanied locally by a similar displacement in γ (yellow arrow), whereas no significant displacements are apparent in δ.

**Unwin and Fujiyoshi, 2012 Supplemental Data**

**
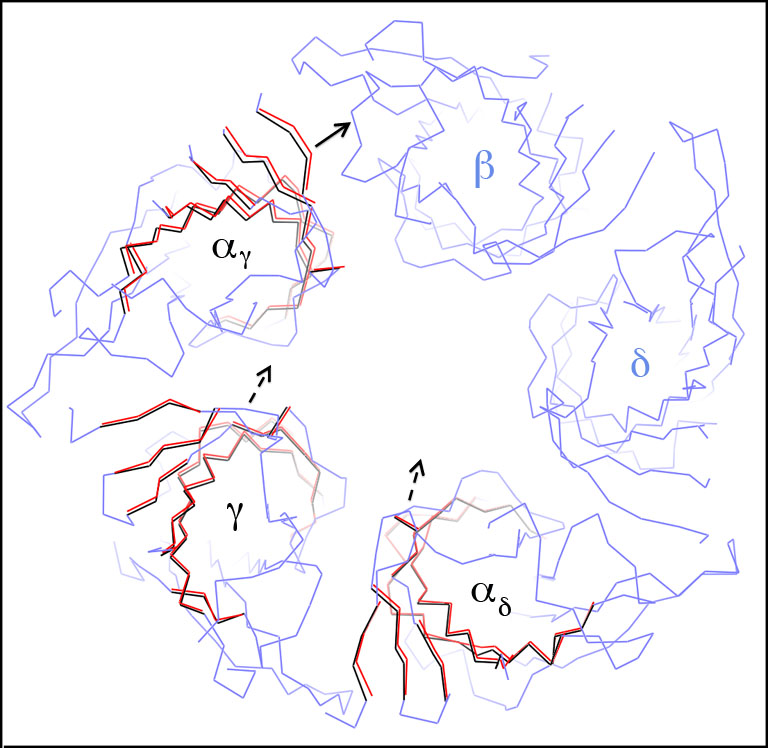
**

**Figure S6.** Coordinated displacements of α_δ_, γ and α_γ_ around the pentamer, shown by superposition of their inner β-sheets (black, closed; red, open). While displacement of the inner sheet of α_γ_ (full arrow) is mainly responsible the outward movement of the β, displacements involving γ and α_δ_ (broken arrows) also appear to contribute in a coordinated way. The cross-section through the pentamer (blue) is from the level of the acetylcholine binding sites to the base of the ligand-binding domain.

**Unwin and Fujiyoshi, 2012 Supplemental Data**


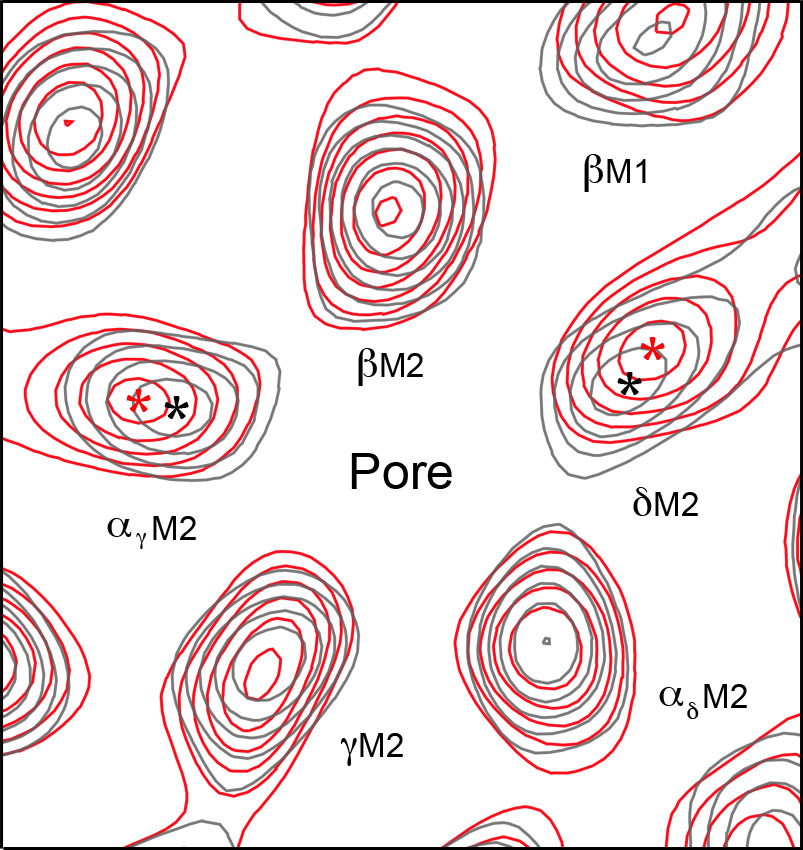


**Figure S7.** Crevice opened up between δM2 and α_δ_M2 shown in projected density maps. The contours plot densities in the membrane portion of the receptor projected down the axis of the pore (closed class: grey contours; open class: red contours). Straightening of δM2, to open the pore, displaces the corresponding density peak (black to red asterisk) a further 1.7Å from α_δ_M2 in a direction almost tangential to the pore axis. The shift in peak location reflects the increased separation of the helix backbones (Fig. 9e). Note that there is a similar peak displacement associated with the straightening of α_γ_M2, but that in this case the direction is radial.

**Unwin and Fujiyoshi, 2012 Supplemental Data**


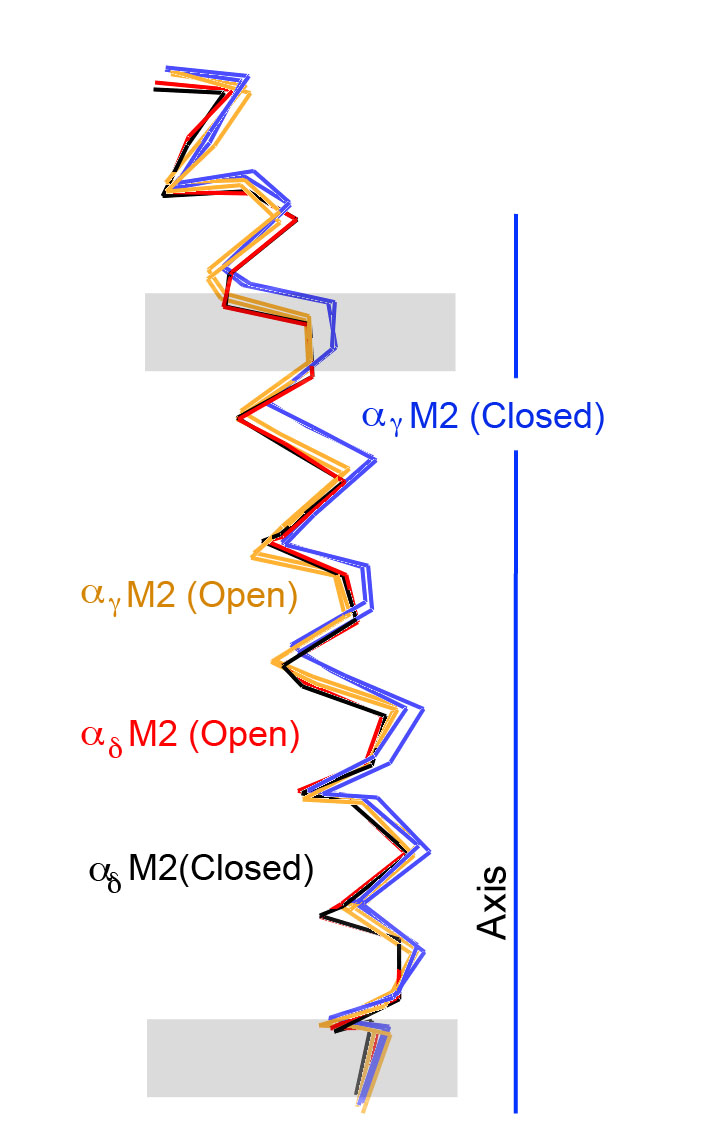


**Figure S8.** Superposition of the α_δ_ and α_γ_ M2 helices. Shown are the two sets of Cα backbones determined for α_γ_M2, based on independent half-data sets (as in Fig. 9b; blue traces: closed class, orange traces: open class), and the aligned M2 helices of α_δ_ (black trace: closed class; red trace: open class). Both α_δ_M2 helices match closely the straight open-class α_γ_M2 helices, indicating that α_δ_M2 has the straight helix conformation in both structures.

**References**

S1. Berriman, J. & Unwin, N. Analysis of transient structures by cryo-microscopy combined with rapid mixing of spray droplets. (1994). *Ultramicroscopy*, **56**, 241-252.

S2. Wang, J.H. & Miller, S. Tracer-diffusion in liquids. II. The self-diffusion as sodium ion in aqueous sodium chloride solutions. (1952). *J. Am. Chem. Soc*. **74**, 1611-1612.

S3. Krnjevic, K. & Mitchell, J.F. Diffusion of acetylcholine in agar gels and in the isolated rat diaphragm. (1960). *J. Physiol.* **153**, 562-572.

S4. Crank, J. *The mathematics of diffusion*. p.29. Oxford, 1956.

S5. Böttcher, B., Wynne, S.A. & Crowther, R.A. Determination of the fold of the core

protein of hepatitis B virus by electron cryomicroscopy. (1997). *Nature* **386**, 88-91.

**Videos**

**Supplementary Movie 1.** Rocking motion between the extracellular and membrane portions of the β subunit.

**Supplementary Movie 2.** Flexure of α_γ_M2 and δM2 affecting dimensions of the pore.

**Supplementary Movie 3.** Membrane domain showing asymmetric movements of βM2, α_γ_M2 and δM2 to widen the pore.
