# Supplementary figures and images for: Gating Movement of Acetylcholine Receptor Caught by Plunge-Freezing
Source: J Mol Biol. 2012 Oct 5;422(5):617–34. doi: 10.1016/j.jmb.2012.07.010 (PMC3443390; doi:10.1016/j.jmb.2012.07.010)

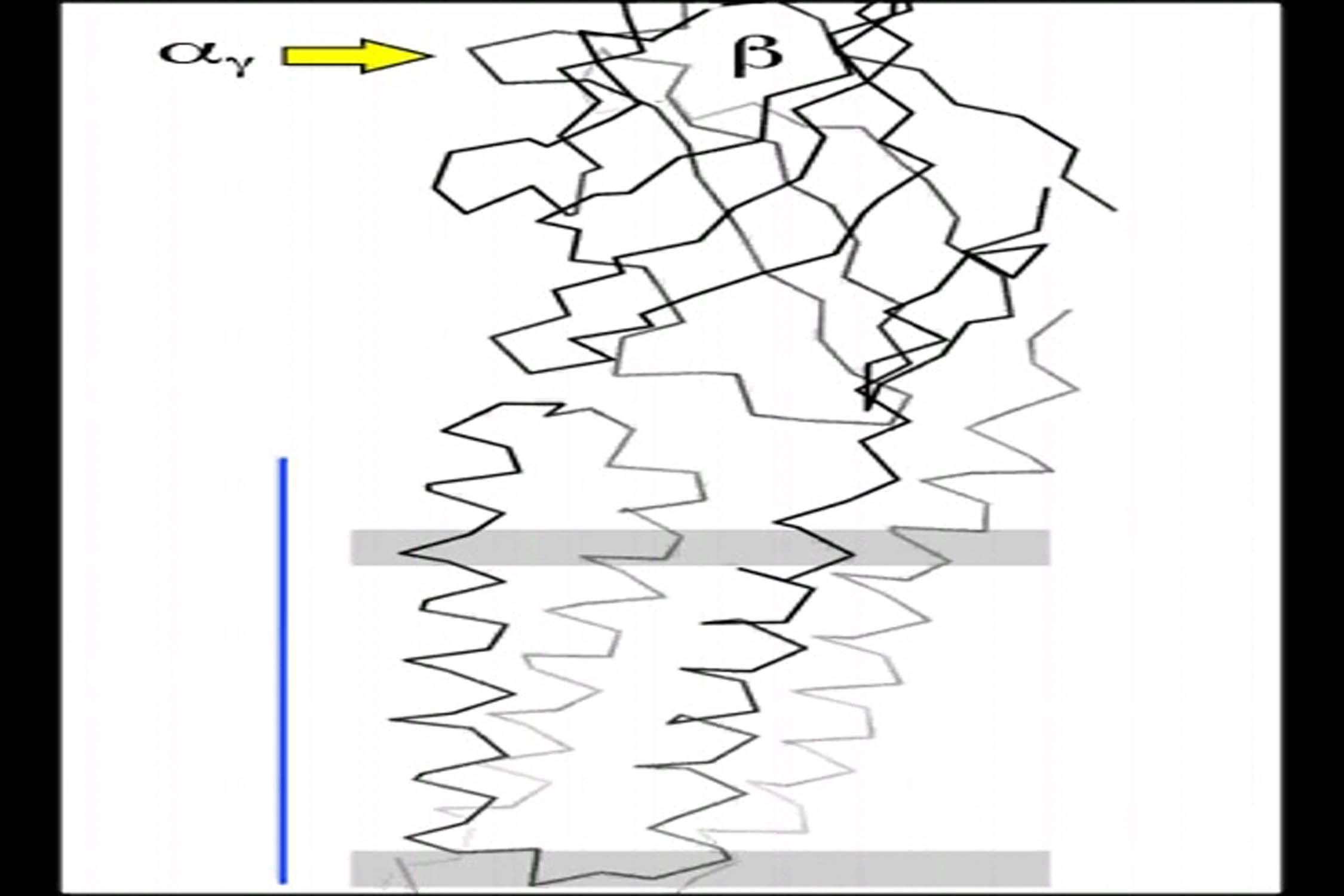

Supplement: Supplementary file 2 — Supplementary Movie 2 [file mmc2.jpg]

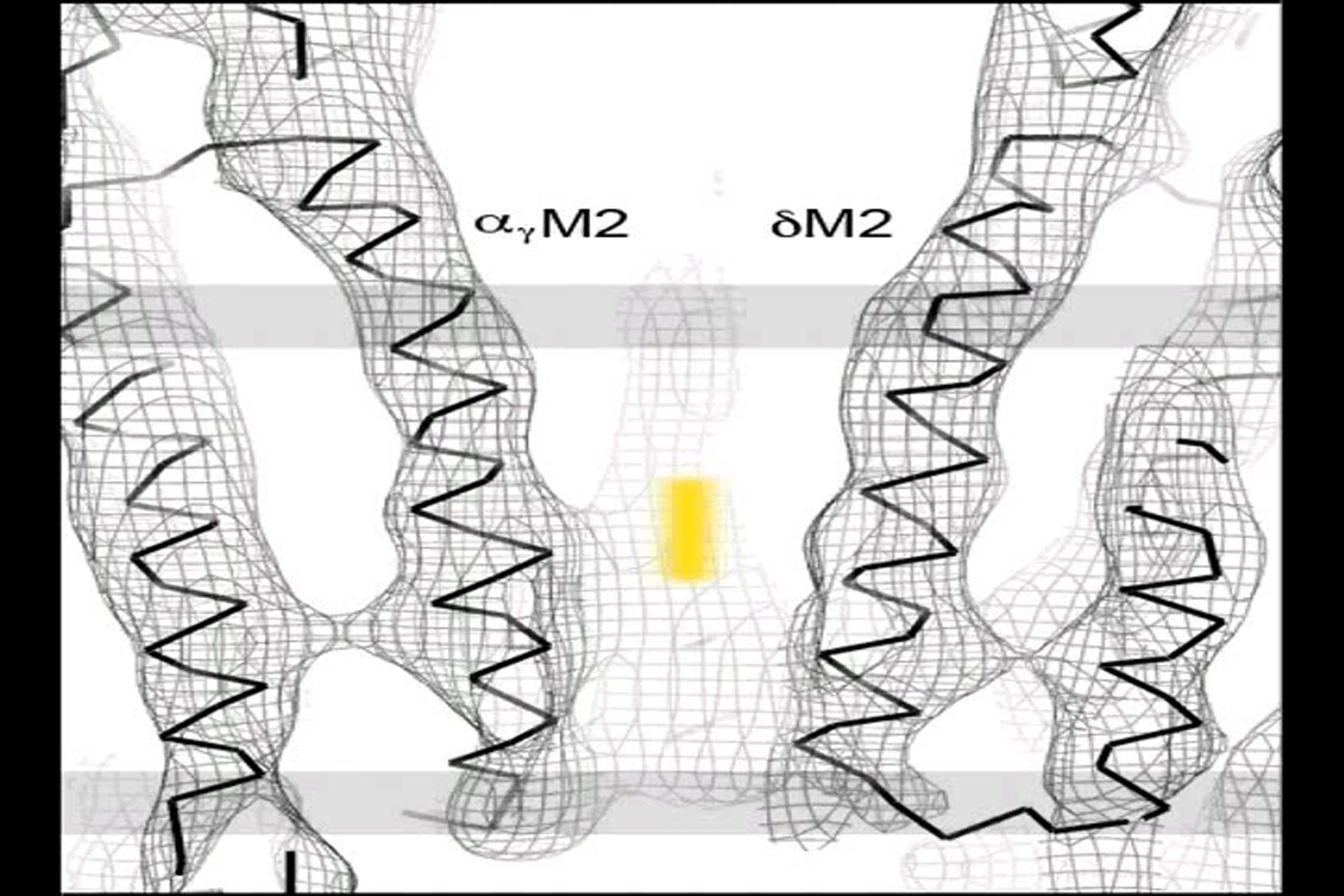

Supplement: Supplementary file 3 — Supplementary Movie 3 [file mmc3.jpg]

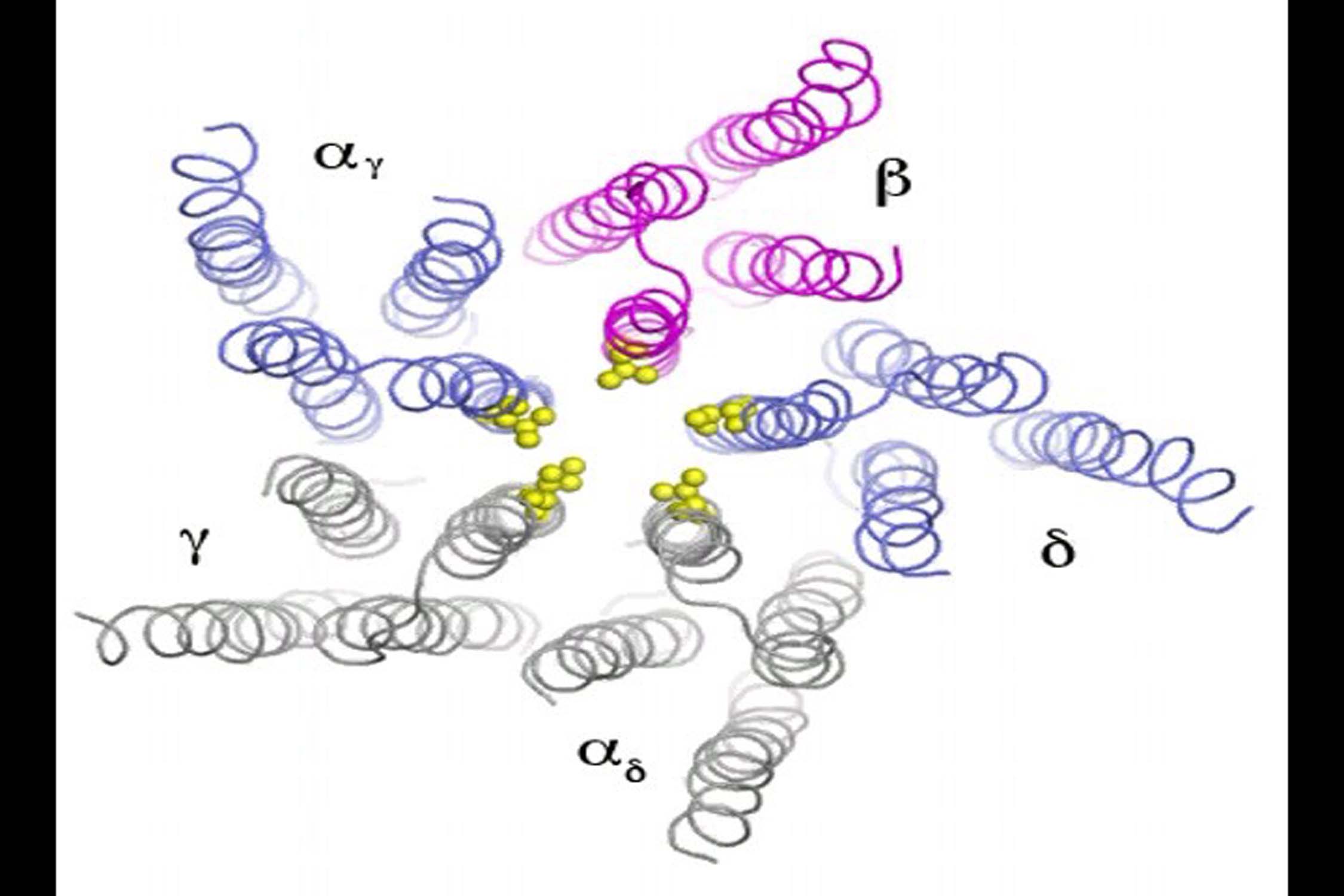

Supplement: Supplementary file 4 — Supplementary materials [file mmc4.jpg]
